# Dairy Products Intake and Endometrial Cancer Risk: A Meta-Analysis of Observational Studies

**DOI:** 10.3390/nu10010025

**Published:** 2017-12-28

**Authors:** Xiaofan Li, Jing Zhao, Peiqin Li, Ying Gao

**Affiliations:** Key Laboratory of Nutrition and Metabolism, Institute for Nutritional Sciences, Shanghai Institutes for Biological Sciences, University of Chinese Academy of Sciences, Chinese Academy of Sciences, 320 Yueyang Road, New Life Science Building, Room A1926, Shanghai 200031, China; xfli@sibs.ac.cn (X.L.); Zhaojing1006@126.com (J.Z.); pqli@sibs.ac.cn (P.L.)

**Keywords:** dairy products, endometrial cancer, nutrients, subgroup analysis, milk, butter, meta-analysis

## Abstract

Observational studies have suggested inconsistent findings on the relationship between dairy products intake and endometrial cancer risk. This study aimed to conduct a meta-analysis to evaluate this correlation; moreover, databases including PubMed, ISI Web of Science, and Embase were screened for relevant studies up to 26 February 2017. The inverse variance weighting method and random effects models were used to calculate the overall OR (odds ratio) values and 95% confidence interval (CI). A total of 2 cohort study and 16 case-control studies were included in the current analysis. No significant association was observed between endometrial cancer risk and the intake of total dairy products, milk, or cheese for the highest versus the lowest exposure category (total dairy products (14 studies): OR 1.04, 95% CI: 0.97–1.11, *I*^2^ = 73%, *p* = 0.000; milk (6 studies): 0.99, 95% CI: 0.89–1.10, *I*^2^ = 0.0%, *p* = 0.43; cheese (5 studies): 0.89, 95% CI: 0.76–1.05, *I*^2^ = 39%, *p* = 0.16). The only cohort study with a total of 456,513 participants reported a positive association of butter intake with endometrial cancer risk (OR = 1.14; 95% CI = 1.03–1.26, *I*^2^ = 2.6%, *p* = 0.31). There was a significant negative association of dairy products intake and endometrial cancer risk among women with a higher body mass index (BMI) (5 studies, OR 0.66, 95% CI = 0.46–0.96, *I*^2^ = 75.8%, *p* = 0.002). Stratifying the analyses by risk factors including BMI should be taken into account when exploring the association of dairy products intake with endometrial cancer risk. Further well-designed studies are needed.

## 1. Introduction

According to the GLOBOCAN 2012, endometrial cancer is the fifth most common cancer in women over the world [1]. Identifying risk factors for endometrial cancer may help to establish prevention strategies and improve life quality. According to the Global Burden of Disease Study 2015, dietary factors are the leading risk factor for chronic diseases, including cancer [2].

Dairy products have been considered part of a healthy diet, and it was recommended to have three cups of dairy products daily, according to the MyPlate guide released by the US Department of Agriculture (USDA) [3]. Dairy products contain measurable amounts of conjugated linoleic acid (CLA) and calcium. CLA was suggested to induce apoptosis of cancer cells [4], and calcium may act with the help of vitamin D (e.g., calcium intake was suggested to be a significant determinant of serum 25OH-D [5]) as a nutrient with antineoplastic potential for cancer prevention [6,7]. The World Cancer Research Fund- The American Institute for Cancer Research (WCRF-AICR) Colorectal cancer 2011 Report [8] showed that milk and calcium could probably decrease colorectal cancer risk. Another meta-analysis illustrated a decreased breast cancer risk with high and moderate intakes of dairy products [9]. The WCRF-AICR Endometrial cancer 2013 Report [10] showed no conclusion concerning the association between total dairy products consumption and endometrial cancer risk based on a systematic literature review in 2007 [11]. Since 2007, two cohort studies and three case-control studies evaluating the association of dairy intake and endometrial cancer were reported. As we all know, the ingredients in whole milk, low-fat milk, cheese, yogurt, and butter differ a lot but the association of milk, cheese, and butter with endometrial cancer risk was not assessed in the meta-analysis in 2007. We aimed to conduct a meta-analysis to explore the association of different dairy products with endometrial cancer risk, including total dairy products, milk, cheese, and butter.

## 2. Materials and Methods

### 2.1. Search Strategy

This meta-analysis was planned, conducted, and reported according to the Preferred Reporting Items for Systematic Reviews and Meta-Analyses (PRISMA) recommendation [12]. PubMed, ISI Web of Science, and Embase were screened for relevant articles up to 20 January 2017. Reference lists of the retrieved articles and previous systematic reviews assessing the association between dairy products intake and the risk of endometrial cancer were manually searched. 

According to the WCRF Specification Manual (available at http://www.wcrf.org), the general search terms of exposure for PubMed included diet [tiab] OR diets [tiab] OR dietetic [tiab] OR dietary [tiab] OR eating [tiab] OR intake [tiab] OR nutrient* [tiab] OR nutrition. The specific keywords about dairy products included “dairy [tiab]” OR “milk [tiab]” OR “yogurt [tiab]” OR “cheese [tiab]”. For the outcome terms, they were consistent with terms published in a previous meta-analysis [11] including: (1) endometrial neoplasm [MeSH]; (2) malign* [tiab] OR cancer* [tiab] OR carcinoma* [tiab] OR tumor* [tiab] OR tumour* [tiab]; (3) endometr* [tiab] OR corpus uteri [tiab] OR uterine [tiab]; (4) #2 and #3; (5) #1 OR #4 [13].

### 2.2. Study Selection

**Figure 1:**
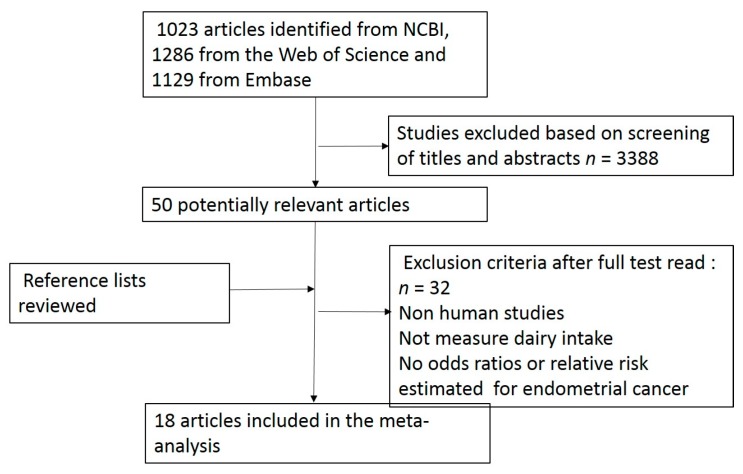
Study flow diagram.

Eligible studies should meet the following criteria: (1) the study design was a case-control study or a prospective study; (2) the exposure included dairy products, milk, yogurt, butter, and cheese; (3) the outcome was endometrial cancer; (4) the odds ratio (OR) or relative risk between the intake of dairy products and the risk of endometrial cancer was reported. We identified relevant 1023 articles from our search on PubMed, 1286 studies from the ISI Web of Science, with literature of Editorial, case report, review, book, news, reference material, meeting, clinical trial, patent, biography, letter excluded, and 1129 relevant articles from our search on Embase. Titles and abstracts were reviewed independently, and fifty pertinent articles were retrieved (Figure 1). After going through the full texts, eighteen articles were considered for the meta-analysis [14,15,16,17,18,19,20,21,22,23,24,25,26,27,28,29,30,31]. Among the eighteen articles, there was one pooled article including another single study conducted by Bravi, who reported odds ratios (ORs) and confidence intervals (CIs) of milk, yogurt, and cheese separately rather than all together as total dairy [26]. Therefore, either of the two articles (not both) was included in the specified meta-analysis. In addition, there was a cohort study including two different study populations, the European Prospective Investigation into Cancer and Nutrition (EPIC) study and the Nurses’ Health Studies NHS I and NHSII conducted in the US [29]. Another study conducted by Ganmaa [28] also included the NHS I in the population, but the concerned outcome in the study was invasive adenocarcinoma. In total, 18 articles [14,15,16,17,18,19,20,21,22,23,24,25,26,27,28,29,30,31] were included in the final analysis.

### 2.3. Data Extraction and Assessment of Study Quality

Data extraction forms for recording study characteristics, quality issues, and results of studies were developed to get as much details of the studies included as possible. Two investigators (Li, X.F. and Zhao, J.) independently extracted the details of the study design, including population, exposure, outcome assessment, sample size, participant characteristics (age, exclusion of hysterectomy, body mass index (BMI), menopause status), time frame, survey method, and statistical analyses, as well as confounders for adjustment from the included studies (Table 1). The Newcastle—Ottawa Quality assessment scale (NOS) was applied to evaluate the quality of the included studies. A study could be awarded a maximum of nine stars. A maximum of two stars could be given for comparability, one for age-matched or adjusted analysis in the study, and one for BMI- and total energy intake-adjusted analysis.

### 2.4. Statistical Analysis

According to the WCRF criteria, the exposures evaluated in more than two cohorts or five case-control studies were used for analysis. There was a sufficient number of studies to conduct a meta-analysis for total dairy and milk intake. Seven studies [14,15,18,19,25,26,29] only reported different dairy items, and we pooled the odds ratios for different dairy items weighted by inverse of the variance within each study [32]. Although there were not enough studies for yogurt and cheese, we also conducted an exploratory meta-analysis of yogurt and cheese to get a general idea of the relation between these products and the risk of endometrial cancer. A random effects model using the method of DerSimonian & Laird was performed to calculate the summary estimate and 95% confidence interval (CI).

The heterogeneity across the studies was estimated using the Mantel–Haenszel model, i.e., if the *p*-value was less than 0.05 in the Q test, or the I-squared was more than 50%, heterogeneity was considered statistically significant, and the random effect model was used for further analysis. Moreover, to search for potential sources of heterogeneity, subgroup analyses were performed by geographic region, age, BMI, and menopausal status. Concerning the lack of the age- and BMI-based group-specific data on endometrial cancer risk, the median or mean value of age and BMI among controls were used to divide the studies to two relative groups. The publication bias was evaluated with funnel plots and the Egger’s regression asymmetry test, i.e., if the *p*-value was less than 0.05, this indicated a significant publication bias. Furthermore, to investigate the impacts of individual studies on the overall results, we also performed a sensitivity analysis by recalculating the overall OR value after removing each study one at a time. 

All statistical analyses were performed with STATA version 12.0 (StataCorp, College Station, TX, USA). All statistical tests were two-sided. 

## 3. Results

### 3.1. Study Characteristics

For the current meta-analysis, we included two cohort studies which included two populations, seven population-based case-control studies, eight hospital-based case-control studies, and one pooled analysis of three Italian hospital-based case-control studies. The population in the study conducted by Filomeno et al. [30] included the same population as in the study conducted by Bravi et al. [26]. Detailed characteristics of the studies are shown in Table 1. Among the 16 case-control studies, 8 were conducted in North America [14,15,16,19,20,21,24,27], 6 in Europe (Bravi, F. et al.; Filomeno, M. et al. both included) [17,22,23,26,30,31], and 2 in Asia [18,25]. In the cohort studies, one population was from the NHS study in the US, and another was from EPIC in Europe. 

### 3.2. Total Dairy Intake and Endometrial Cancer Risk

**Figure 2:**
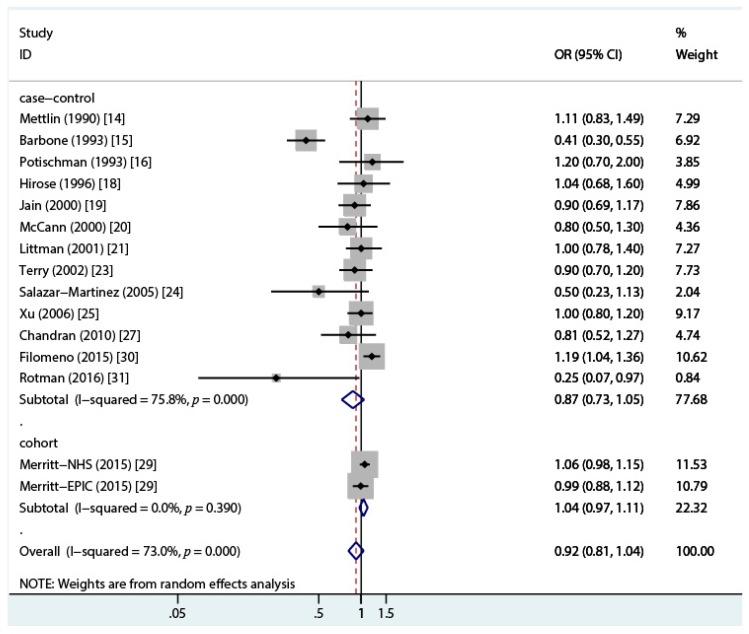
Forest plot of the summary risk estimate of endometrial cancer in the highest category of dairy intake compared with that in the lowest category by the random effects model. OR, odds ratio; CI, confidence interval.

**Figure 3:**
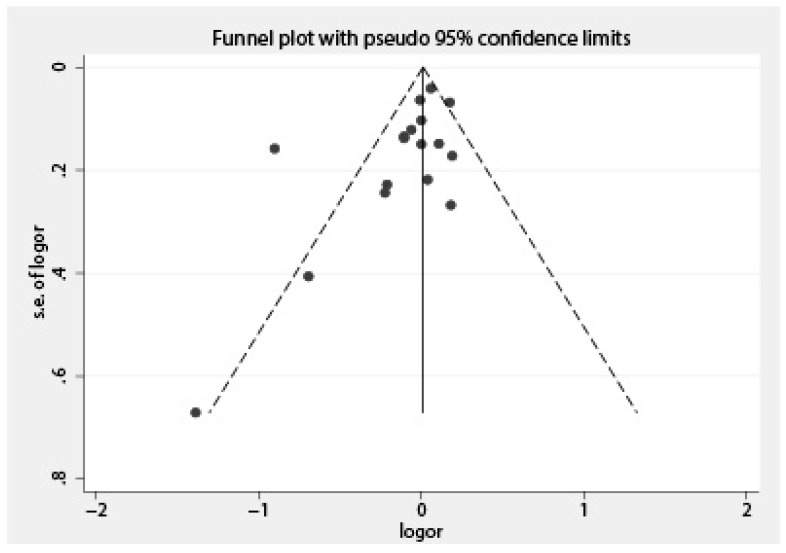
Funnel plot of the meta-analysis for the association between total dairy intake and risk of endometrial cancer.

**Figure 4:**
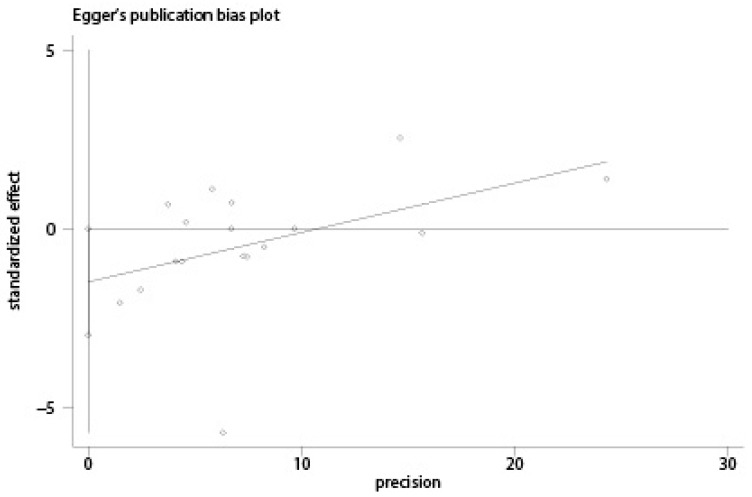
Egger’s regression asymmetry test of the meta-analysis for the association between total dairy intake and risk of endometrial cancer.

**Figure 5:**
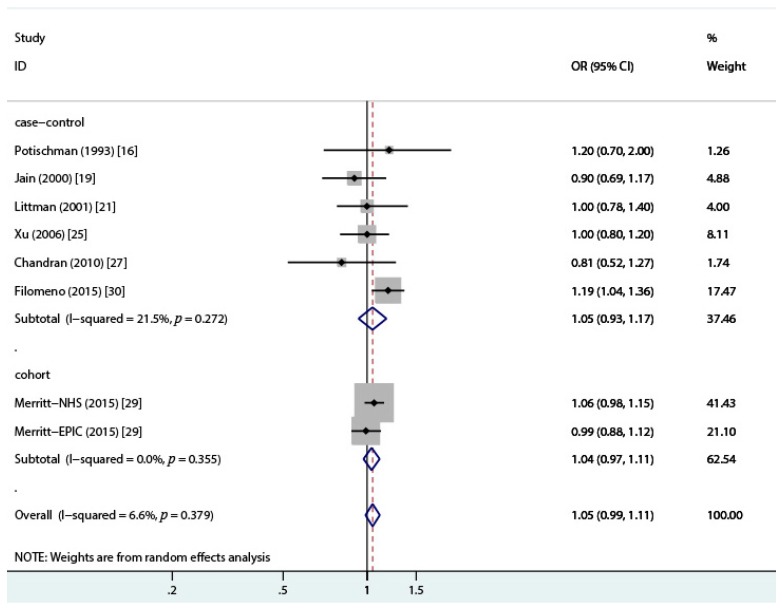
Forest plot of the summary risk estimate of endometrial cancer in the highest category of total dairy intake compared with that in the lowest category after exclusion of Newcastle—Ottawa Quality assessment scale (NOS) scores less than 6 by the random effects model. OR, odds ratio; CI, confidence interval.

A total of 10 case-control studies [16,17,20,21,22,23,24,27,30,31] reporting the association between total dairy intake and endometrial cancer risk were included in the current analysis. Seven studies [14,15,18,19,25,26,29] which only reported a specified type of dairy products were also included in the overall analysis. We pooled the odds ratios for different dairy items weighted by the inverse of the variance within each study [32]. As Petridou et al. [22] and Tzonou et al. [17] only reported the OR per quantile of dairy intake (1.21 (95% CI = 0.86–1.69) and 0.94 (95% CI = 0.74–1.19), respectively), the total studies included in the following analyses were 13 case-control [14,15,16,18,19,20,21,23,24,25,26,27,30,31] and 2 cohort studies (reported in a single paper) [29]. The overall OR was 0.92 (95% CI = 0.81–1.04, *I*^2^ = 73%, *p* = 0.000) between total dairy intake and endometrial cancer risk when comparing the highest category of total dairy intake to the lowest category. Based on the 13 case-control studies, the estimated OR was 0.87 (95% CI = 0.73–1.05, *I^2^* = 75.8%, *p* = 0.000), and the OR for the cohort studies was 1.04 (95% CI = 0.97–1.11, *I*^2^ = 0.0%, *p* = 0.39) (Figure 2). Funnel plot and the Egger’s regression asymmetry test for publication bias suggested a significant publication bias (*p* = 0.0499) (Figure 3 and Figure 4), reflecting the relative absence of studies. The sensitivity analysis suggested that the study conducted by Barbone et al. [15] contributed a large amount of heterogeneity (*I*^2^ varied from 75.8% to 35.2% after excluding the study conducted by Barbone et al.). After excluding the studies with NOS stars less than 6, the OR for case-control studies was 1.05 (95% CI = 0.93–1.17, *I*^2^ = 21.5%, *p* = 0.27), and the OR for cohort studies was 1.05 (95% CI = 0.99–1.11, *I*^2^ = 7.7%, *p* = 0.37) (Figure 5). Pooling different food items into a total dairy variable for studies only reporting on specific dairy types might have introduced a new bias but it also provided us with opportunities to conduct a subgroup analysis and a sensitivity analysis and to make full use of the available data. We also conducted an analysis of the association of total dairy products intake and endometrial cancer risk considering those studies that reported the exposure to total dairy products [14,16,19,20,21,23,26,27,29], and the OR was 0.93 (95% CI = 0.76–1.14) for eight case-control studies, 1.26 (95% CI = 0.94–1.69) for invasive adenocarcinoma, and 1.03 (95% CI = 0.64–1.66) for pre-invasive adenocarcinoma in the cohort study conducted by Ganmaa et al. [28].

With the limited amount of studies, we conducted a further stratified analysis using the following parameters: geographic region, BMI, age, and state of menopause. In the analysis stratified by geographical region, the OR for total dairy intake and risk of endometrial cancer was 0.85 (95% CI = 0.68–1.06, *I*^2^ = 80.2%, *p* = 0.000) in North America, 1.01 (95% CI = 0.83–1.22, *I*^2^ = 69.9%, *p* = 0.02) in Europe, and 1.01 (95% CI = 0.84–1.21, *I*^2^ = 0.0%, *p* = 0.87) in Asia. The analysis stratified by BMI showed an OR of 0.66 (95% CI = 0.46–0.96, *I*^2^ = 75.8%, *p* = 0.002) in the group where the median BMI was greater than 25, and 1.05 (95% CI = 0.98–1.13, *I*^2^ = 29.0%, *p* = 0.23) in the group where the median BMI was no more than 25. Additionally, in the subgroup classified by median age, the group older than 55 showed an OR for risk of endometrial cancer of 0.86 (95% CI = 0.67–1.11, *I*^2^ = 83.2%, *p* = 0.000) when comparing the highest category with the lowest category of total dairy intake, whereas the group younger than 55 showed an OR of 1.03 (95% CI = 0.97–1.10, *I*^2^ = 0.0%, *p* = 0.51). When stratified by menopause status (menopause frequency greater or less than 70%), the ORs of dairy intake and endometrial cancer risk were 0.86 (95% CI = 0.70–1.06, *I*^2^ = 0.0%, *p* = 0.88) and 1.06 (95% CI = 0.98–1.14, *I*^2^ = 29.4%, *p* = 0.24), respectively.

### 3.3. Milk Intake and Endometrial Cancer Risk

**Figure 6:**
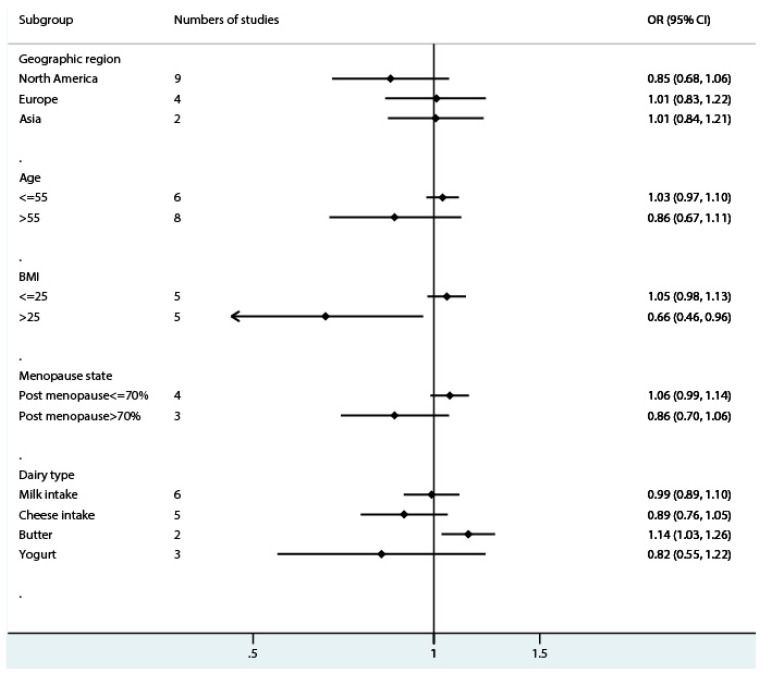
Subgroup analysis of the forest plot of the risk estimate of endometrial cancer comparing the highest category of dairy intake with the lowest category. OR, odds ratio; CI, confidence interval; BMI, body mass index.

There were six studies [14,15,18,19,25,29] that reported the association between milk intake and endometrial cancer risk. No significant association was found between milk intake and endometrial cancer risk (five case-control studies: OR = 1.00; 95% CI = 0.86–1.16, *I*^2^ = 16.4%, *p* = 0.31; one cohort study: OR = 0.97; 95% CI = 0.81–1.16, *I*^2^ = 0.0%, *p* = 0.43). The overall OR was 0.99 (95% CI = 0.89–1.10, *I*^2^ = 0.0%, *p* = 0.43) (Figure 6).

### 3.4. Cheese Intake and Endometrial Cancer Risk

The pooled estimate of three case-control studies [15,19,26] showed that cheese was not associated with endometrial cancer risk (OR = 0.81, 95% CI = 0.53–1.24, *I*^2^ = 59.5%, *p* = 0.09) (Figure 6). No significant association (RR = 0.91, 95% CI = 0.77–1.07, *I*^2^ = 37.4%, *p* = 0.206) was observed also for the two cohort studies.

### 3.5. Butter Intake and Endometrial Cancer Risk

Only the two cohort studies [29] tested the association between butter intake and endometrial cancer risk. The overall OR for the two populations was 1.14 (95% CI = 1.03–1.26, *I*^2^ = 2.6%, *p* = 0.31) (Figure 6).

## 4. Discussion

In this meta-analysis, no significant association between total dairy products intake and endometrial cancer risk was observed. Though geographic region, BMI, age, and state of menopause did not modify the association significantly, we observed a relatively lower risk of endometrial cancer associated with dairy product intake in women with a higher BMI (Figure 6), and this is probably no more than chance variation. The amount of studies among these groups was limited. With a lack of original data, the subgroup analyses for age and BMI were carried out by dividing the studies into groups according to the median value of the controls; the power of the stratified analysis based on these values was also limited. Though we did not observe a significant association of endometrial cancer risk with milk and cheese, increased cancer risk was suggestively associated with butter intake, even if this result was based on only two studies.

The exposure to dairy products was not considered in the Endometrial Cancer 2013 Report and its effect remained limited with no conclusions, based on the meta-analysis conducted in 2007 [11]. Compared with the meta-analysis in 2007, we included 11 more studies [14,15,18,19,25,26,27,28,29,30,31]. However, we also did not found a significant association between the overall intake of dairy products and endometrial cancer risk. Among the newly included studies [14,15,18,19,25,26,27,28,29,30,31], only three studies reported significant results, two [15,31] of them reported an inverse association of total dairy with endometrial cancer risk, and one [30] reported a positive association of total dairy intake with endometrial cancer risk. The opposite associations of these three studies [15,30,31] might be due to the following reasons: (1) small sample size that could result in a large variance of the results. The sample size in the study conducted by Barbone et al. [15] and Rotman et al. [31] was less than 200 which limited the statistical power to detect a moderate difference; (2) various dietary patterns between the study populations: the populations of the three studies [15,30,31] reporting significant results came from different countries, and different dietary patterns consumed in the three countries might have led to different results. Barbone et al. [15], Rotman et al. [31], and Filomeno et al. [30] separately reported results concerning the American, the Polish, and the Italian populations. The consumption of low-fat or skim milk instead of whole milk was increased during the period from 1970 to 1992 in the US [33], which might somehow explain the inverse association by Barbone et al. [15]. As for the negative association suggested by Rotman et al. [31], the Polish were reported to consume less butter [34], which was suggested to be associated with higher endometrial cancer risk. The Italian mainly consumed cheese [35] containing more saturated fatty acids that were reported to increase endometrial cancer risk [13], which is consistent with the positive association suggested by Filomeno et al. [30]. However, our meta-analysis did not observe an obvious difference depending on the type of the consumed dairy products. 

Dairy products contain saturated fatty acids and estrogen which were reported to be positively associated with endometrial cancer risk [13,36]. High saturated fat consumers were reported to suffer from reduced insulin sensitivity, which is a risk factor of endometrial cancer [37]. Estrogens could mediate cellular growth and differentiation in the endometrial tissue [36]. On the other hand, several nutrients in dairy products might inhibit carcinogenesis. Dairy products are an excellent source of calcium [38] which was reported to be significantly associated with reduced endometrial cancer risk [6,38]. Calcium in dairy products may be a potential cancer prevention element through vitamin D, as it is highly correlated and metabolically tied to vitamin D [6]. Vitamin D might reduce the cell expression of osteopontin and increase E-cadherin to reduce endometrial cancer development [39]. Dairy products are also an excellent source of conjugated linoleic acid [40] which was reported to be significantly associated with reduced endometrial cancer risk. Specifically, Cis-9, trans-11 conjugated linoleic acid (CLA) can even induce apoptosis of endometrial cancer cells [5]. Dairy products are also an important source of odd-chain saturated fatty acids (C15:0, C17:0), which were reportedly linked to a decreased risk of coronary heart disease (CHD) and low-grade prostate cancer [41,42,43]. However, how the different nutrients work together, and when dairy products intake is protective versus harmful for endometrial cancer risk still needs further research. 

Indeed, the ingredients in whole milk, low-fat milk, cheese, yogurt, and butter differ a lot. Butter contains more fat while cheese contains more calcium. When we explore whether dairy products intake is protective or harmful for endometrial cancer, we need to consider the varying ingredient content in different dairy products. Furthermore, no significant association was found between endometrial cancer risk and milk intake, as well as cheese intake in the current study. Only the two cohort studies [29] reported a positive association between butter intake and endometrial cancer risk, which might be due to the high content of saturated fat. However, this needs further study in other prospective studies.

This meta-analysis has several strengths. The sensitivity analyses were conducted to figure out the influence of each study on the summary of the estimates. This is the first time that the potential effects of modifications of the dairy intake on endometrial cancer risk are evaluated by geographic region, age, BMI, and menopausal status. Limitations of this meta-analysis should be noted too. Firstly, the number of studies eligible for the meta-analysis was limited, and 16 out of 18 reports were case-control studies, which were susceptible to recall a bias. In addition, the sample size in six case-control studies [15,17,18,22,24,31] was lower than 200 which limited the statistical power. Secondly, dairy product intake levels differed widely between the studies, which might have introduced a significant heterogeneity due to the fact that the reference value varied a lot in the different studies. For instance, Barbone et al. [15] compared a group with a dairy intake of more than once a month to another group with an intake lower than once a month, while Mettlin et al. [14] compared a group with a daily dairy intake to another group with no dairy intake. Notably, individuals that never or seldom consumed dairy products might be lactose intolerant or allergic to dairy products. They may be genetically different from those who consume more dairy products. In addition, they might be exposed to different dietary products (e.g., milk alternatives) which could bias the results if they were used in the reference group, preventing the identification of the effects of high dairy intake in the other groups. Thirdly, not all the studies considered the intake of both total and specific types of dairy products. Fourthly, none of the studies specified the subtypes of endometrial cancer, and this might also induce heterogeneity. Type I endometrial cancer is estrogen-dependent and associated with endometrial hyperplasia, whereas type II endometrial cancer is estrogen-independent and associated with endometrial atrophy [44]. 

## 5. Conclusions

In summary, this meta-analysis did not observe a statistically significant association between overall dairy products intake and endometrial cancer risk. However, increased cancer risk was suggestively associated with butter intake, even if this result was based on only two studies. There was a significant negative association of dairy products intake and endometrial cancer risk among women with a higher BMI. Future well-designed prospective studies with precise measurements of dairy products exposure with biomarkers, data from various continents, and stratified analyses by risk factors including BMI are needed to evaluate the effect of different types of dairy products on endometrial cancer risk.

## Figures and Tables

**Table 1:** Characteristics of the included studies evaluating dairy products and endometrial cancer risk.

Reference	Geographic Region	Case/Control (Cohort)	Age (Case)	Exclusion of Hysterectomy	Time Frame for Dietary Assessment ^§^	Dairy Source	OR (95% CI)	Covariates^ ‖^	NOS Stars
**Cohort**									
Merritt, 2015 [29]	Europe	1303/301,107	25–70	yes	dietary questionnaire EPIC (1992–2000)	Butter: >10.3 vs. 0 g/day Yogurt: >145.3 vs. 0 g/day Cheese: >74.7 vs. 0 g/day Milk: >292.7 vs. 0 g/day	1.23 (1.03, 1.47) 1.15 (0.98, 1.36) 0.83 (0.69, 1.01) 0.97 (0.81, 1.15)	B.E.S.H.R	8
Merritt, 2015 [29]	US	1531/205,863	30–55/25–42	yes	every 4 years NHS I 1976 (1980–2010) NHSII1989 (1991–2011)	Butter: 5.0 vs. 0 g/day Cheese: 28.0 vs. 2 g/day Yogurt: 105.4 vs. 0 g/day	1.10 (0.97, 1.24) 0.98 (0.82, 1.16) 1.06 (0.93, 1.22)	B.E.S.H.R	7
**Population-based Case-control**									
Potischman, 1992 [16]	US	399/296	20–74	yes	few years	Dairy: 17.6 vs. 6.0 times/week	1.2 (0.70, 2.00)	A.B.E.H.S.R	7
McCann, 2000 [20]	US	232/639	40–85	yes	2 years	Dairy: >56 vs. ≤32 times/month	0.8 (0.5, 1.3)	A.B.H.S.R	6
Jain, 2000 [19]	Canada	552/562	30–79	yes	-	Milk: 413 vs. 84 g/day Cheese: 29.1 vs. 5.3 g/day	0.86 (0.59, 1.24) 0.94 (0.65, 1.37)	A.B.E.H.S.R	7
Littman, 2001 [21]	US	679/944	45–74	yes	5 years	Dairy: >2.4 vs. <1.2 servings/day	1.0 (0.78, 1.40)	A.B.E.H.S	8
Terry, 2002 [23]	Sweden	709/2887	50–74	yes	1 year	Dairy: median intake 35 vs. 5 serving/week	0.90 (0.70, 1.20)	A.B.S	6
Xu, 2006 [25]	China	1204/1212	30–69	yes	5 years	Milk: ever vs. never	1.0 (0.80, 1.20)	A.B.E	7
Chandran et al., 2010 [27]	US	417/395	>21	yes	6 months	Dairy: ≥0.99 vs. <0.26 cups/day/1000 kcal)	0.81 (0.52, 1.27)	A.B.E.S.H.R	7
**Hospital-based Case-control**									
Mettlin, 1990 [14]	US	231/1300	19–97	-	habits before	Whole Milk: daily vs. none 2% Milk: daily vs. none Skim milk: daily vs. none	1.50 (1.0, 2.40) 1.0 (0.7, 1.40) 0.90 (0.50, 1.50)	A.S	5
Barbone, 1993 [15]	US	103/236	mean 64	yes	1 year	Skim milk: ≥1 vs. <1/month sour cream: ≥1 vs. <1/month yogurt: ≥1 vs. <1/month cheese: ≥1 vs. <1/month	0.60 (0.30, 1.0) 0.40 (0.2, 0.70) 0.30 (0.20, 0.60) 0.40 (0.20, 0.90)	A.E.H.S.R	5
Tzonou, 1996 [17]	Greece	145/298	-	not mention	1 year	Dairy: per quartile	0.94 (0.74, 1.19)	A.B.E.H.S.R	6
Hirose, 1996 [18]	Japan	145/26,751	>20	not mention	-	Milk: daily vs. occasional/none	1.04 (0.68, 1.60)	B.S.R	5
Petridou, 2002 [22]	Greece	84/84	-	yes	12 months	Dairy: per quartile	1.21 (0.86, 1.69)	(A).B.E.R	7
Salazar-Martinez, 2005 [24]	Mexico	85/629	22–79	yes	1 year	Dairy: tertile3 vs. tertile1	0.5 (0.23, 1.13)	A.B.E.R	6
Bravi, 2009 [26]	Italy	454/908	18–79	yes	2 years	Milk and yogurt: >10.50 vs. <1 servings/week cheese: >6.32 vs. <2.4 servings/week	1.24 (0.8, 1.93) 1.24 (0.80, 1.93)	B.E.H.R	6
Filomeno, 2015 [30]	Italy	1411/3668	median 61	-	2 years	Dairy: >median vs. <median	1.19 (1.04, 1.36)	A.B.E.S.H.R	5
Rotman, 2016 [31]	Poland	68/480	40–84	-	-	Dairy: 250 vs. 0 g/day	0.25 (0.07, 0.97)	-	4

^§^ Time before onset of cancer or before interview; ^‖^ Covariates: A = age, B = body mass index (BMI) or weight, E = total energy, H = HRT (hormone replacement therapy) or estrogen use, R = reproductive factors, S = Smoking. (A): matched on age; NOS: Newcastle—Ottawa Scale; OR: odds ratio.
